# Metabolic markers in bipolar disorder with childhood trauma exposure: a systematic review

**DOI:** 10.1017/S1092852926100881

**Published:** 2026-03-24

**Authors:** Hernan F. Guillen-Burgos, Nicolas Montero, Isabela Orozco, Gabriela Gamba, Valentina Vanegas, Adelaida Uribe, Andrea Morelli, Sergio M. Moreno-Lopez, Juan F. Galvez-Florez, Roger S. McIntyre

**Affiliations:** 1 Pontificia Universidad Javeriana, Department of Psychiatry and Mental Health, Bogotá DC, Colombia; 2 Universidad Simon Bolivar, Center for Clinical and Translational Research, Centro de Investigaciones en Ciencias de la Vida (CICV), Barranquilla, Colombia; 3 Center for Clinical and Translational Research, Bogotá DC, Colombia; 4 Pontificia Universidad Javeriana, Faculty of Medicine, Bogotá DC, Colombia; 5 Universidad de los Andes, Faculty of Medicine, Bogotá DC, Colombia; 6 Department of Psychiatry, University of Toronto, Toronto, Ontario, Canada; 7 Department of Pharmacology & Toxicology, University of Toronto, Ontario, Canada

**Keywords:** Bipolar disorder, childhood trauma, metabolic biomarkers, inflammation, cardiometabolic risk

## Abstract

Bipolar disorder (BD) is associated with increased cardiometabolic risk, contributing to elevated morbidity and premature mortality. Childhood trauma (CT) is a common environmental risk factor in BD and may exacerbate metabolic dysfunction, but no prior systematic synthesis has focused on their intersection. The objective of this review was to systematically review and synthesize evidence on the association between childhood trauma exposure and metabolic biomarkers in adults with bipolar disorder. This review adhered to PRISMA 2020 guidelines and was registered in PROSPERO (ID CRD420251045565). A comprehensive search of PubMed/MEDLINE, Web of Science, Scopus, and Embase (from inception to September 2025) was conducted. Eligible studies were peer-reviewed observational studies assessing associations between CT and metabolic markers (eg, BMI, lipids, HbA1c, hs-CRP) in adult BD populations. Data extraction and NIH quality assessments were performed independently by multiple reviewers. Sixteen studies were included (total *n* ≈ 6,200 across study samples). CT was significantly associated with higher body mass index and elevated hs-CRP. Two third of studies reported adverse associations with lipid profiles, and one study showed increased HbA1c among CT-exposed BD patients. Most findings emerged from cross-sectional designs, though one longitudinal study revealed large effect sizes across multiple metabolic markers. CT is consistently associated with adverse metabolic outcomes in individuals with BD, particularly elevated BMI, inflammation, and dyslipidemia. These findings support the need for trauma-informed metabolic screening and personalized interventions in this subgroup BD population. Further prospective studies are warranted to elucidate causal pathways and inform personalized care.

## Introduction

Bipolar disorder (BD) is a chronic mental disorder characterized by recurrent episodes of mania, hypomania, depression, and mixed episodes, with a high burden of morbidity and mortality.^1^ Beyond its affective manifestations, patients with BD have an elevated risk of cardiometabolic diseases, which contributes to a significant reduction in their life expectancy compared to the general population.^2^^,^^3^ This cardiometabolic risk appears to be influenced by multiple factors, including genetic, epigenetic, and environmental factors such as childhood trauma (CT). CT is conceptualized as adverse experiences occurring before age 18, including abuse or neglect (emotional, physical, and sexual abuse; emotional and physical neglect), as operationalized by the validated instruments used in the included studies (eg, the Childhood Trauma Questionnaire [CTQ]).

CT has been widely studied in relation to the onset and clinical course of BD, as well as its comorbidities.^1^^,^^4^ Meta-analytic evidence supports an association between childhood adversity and BD.^5^ Prior studies have also reported that CT exposure is associated with poorer treatment response and suicidal behavior in BD.^6^ For example, in a randomized clinical trial, CT exposure moderated the antidepressant response to adjunctive infliximab in bipolar depression.^7^ Additionally, observational data suggest that a history of CT exposure is associated with a higher prevalence of suicidal behavior in BD.^8^^,^^9^

Recent studies have demonstrated significant associations between BD and a range of metabolic disturbances.^10^ Specifically, metabolic disturbances concerning cholesterol and triglycerides levels, glucose metabolism, body mass index (BMI), and inflammatory markers such as C-reactive protein (CRP).^11^^–^^13^ The interplay among metabolic disturbances and evolving pathophysiology of BD should be of considerable importance, as metabolic biomarkers might elucidate some potential pathways to improve diagnosis and treatment.

Adverse childhood experiences (ACEs), including CT exposure to variants of abuse and/or neglect, have been associated with a variety of psychiatric disorders, including BD.^14^ Moreover, a growing body of research also suggests that CT exposure may lead to changes in emotional and metabolic profiles, and therefore contribute to increased severity and chronicity of BD.^15^^,^^16^ Perhaps, the interplay between CT exposure, metabolisms, and neurobiology underscores the complexity of how CT may influence anticipation, delayed diagnosis, and a more severe course in BD.^17^^–^^19^

At present, relatively few studies have examined the association between CT exposure and the expression of metabolic biomarker profiles in BD. To the best of our knowledge, a qualitative synthesis focused specifically on this clinical association has been lacking. Accordingly, this systematic review aims to provide an updated and comprehensive examination of the association between CT exposure and metabolic biomarker expression in BD.

To guide this systematic review, we formulated our research question using the PECO framework. The population of interest comprised adults diagnosed with BD. The exposure under investigation was a history of childhood trauma (CT), assessed through validated instruments across included studies. The comparator groups included individuals with BD who did not report childhood trauma (CT). The primary outcomes were metabolic biomarkers, including HbA1c, lipid fractions (HDL, LDL, triglycerides), inflammatory markers such as CRP/ high sensitive C-reactive protein (hs-CRP), and body mass index (BMI).

## Methods

### Search strategy

This review follows the 2020 Preferred Reporting Items for Systematic Review and Meta-Analyses (PRISMA) guidelines.^20^ This systematic review was registered on PROSPERO International Prospective Register of Systematic Reviews (ID: CRD420251045565). A comprehensive literature search was conducted using databases including PubMed, Web of Science (Web of Science Core Collection, Current Contents Connect, Grants Index, KCI-Korean Journal Database, Medline, Preprint Citation Index, ProQuest, Dissertations & Theses Citation Index, SciELO Citation Index), Scopus, and Embase. The search string was inserted into each database, where further details can be found in Table S1 in the Supplementary Material. All terms were relevant to bipolar disorder, childhood trauma, and metabolic markers. The literature search occurred from inception to September 30, 2025.

### Metabolic biomarker selection

A predefined list of biomarkers was established prior to the screening phase, based on an extensive review of previous literature and expert consensus in the field of psychiatric–metabolic comorbidity.^21^^–^^23^ These included—but were not limited to—glucose, HbA1c, lipid fractions (HDL, LDL, total cholesterol, triglycerides), inflammatory markers (eg, hs-CRP), and body mass index measure.

### Study selection and eligibility criteria

**Figure 1.**
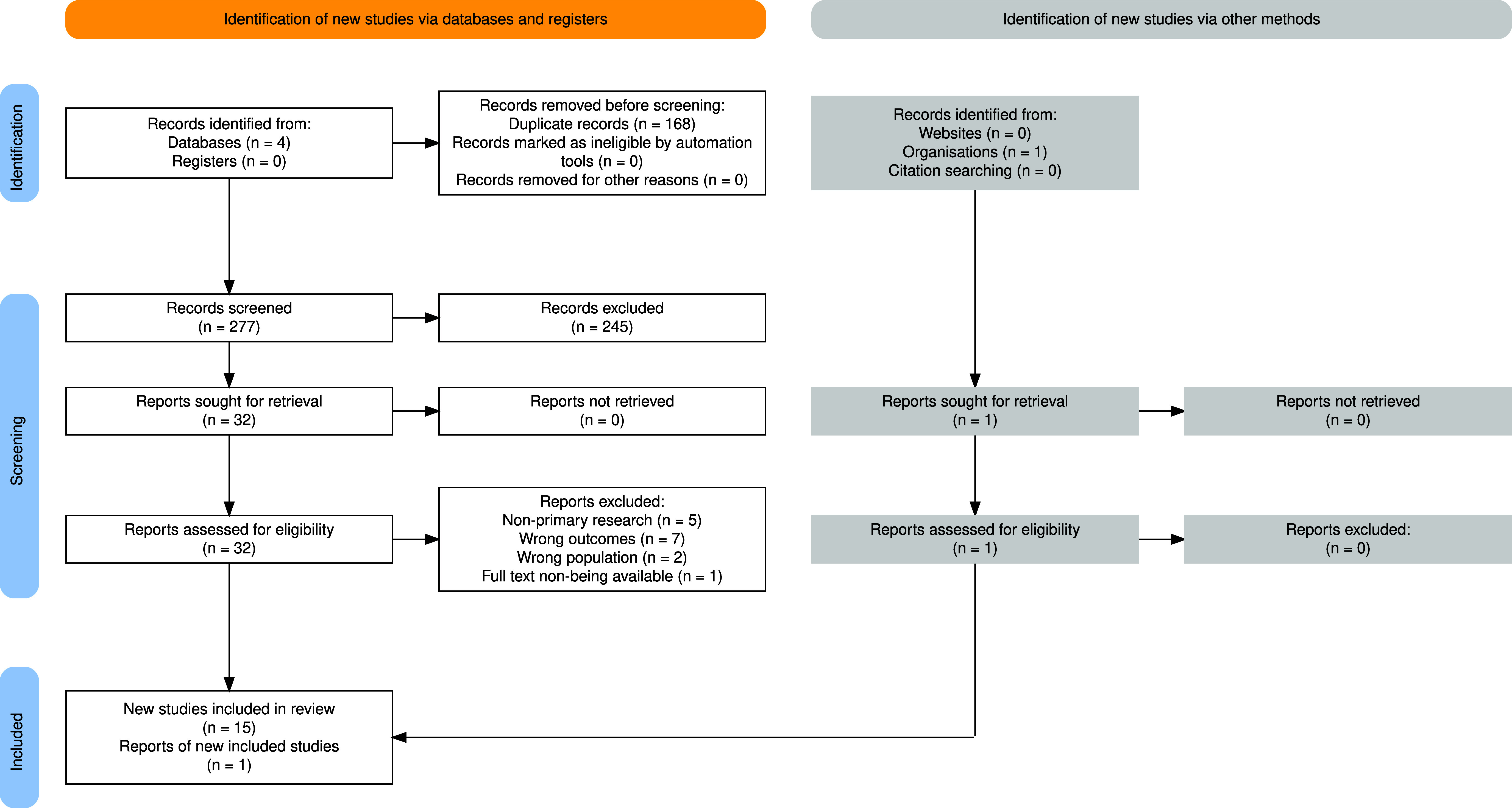
PRISMA Flowchart of included studies

Relevant studies retrieved from the comprehensive search were screened based on the inclusion and exclusion criteria (Table 1). Three independent reviewers (NM, IO, GG) conducted an initial screening of abstracts and titles for potentially relevant studies. Subsequently, full-text screening was performed by the same reviewers. Only articles deemed relevant according to the eligibility criteria, by all reviewers, were included for data extraction and analysis (Figure 1). Any conflicts were resolved between all reviewers or by consulting a fourth reviewer (HGB). The software Rayyan was employed to facilitate the study selection process.^24^ Rayyan automatically eliminated most duplicate studies. Additional duplicates were manually removed.

**Table 1.** Eligibility Criteria

Inclusion criteria	Peer-reviewed primary research articles Observational studies (case–control, cohort, and cross-sectional studies) Population with bipolar disorder with or without mental/physical comorbidity Adult population (age >18 years) only Childhood trauma/childhood maltreatment (abuse and neglect) such as exposure Full-text articles are available online
Exclusion criteria	Non-primary articles (eg, literature reviews, systematic reviews, meta-analyses, posters, abstracts, guidelines, protocols and theses) Studies conducted on animal models (eg, rats, mice) or other non-human populations Non-English language Full-text is not available

Studies were included if they were primary research articles examining the association between CT exposure and metabolic markers (cholesterol, HDL, LDL, triglycerides, glycated hemoglobin, glycemia, hs-CRP, BMI, and blood pressure) in adults (≥18 years) with a primary diagnosis of BD, as defined by DSM-5, DSM-5-TR, DSM-4, DSM-4-TR, or ICD-10 criteria. Additionally, only full-text articles available in English were considered. Studies were excluded if they were secondary articles. Case studies and animal studies were also excluded (Table 1). Childhood trauma exposure was accepted as defined by each study using validated or structured retrospective measures (most commonly the CTQ/CTQ-SF instruments) or equivalent clinician-administered assessments. For studies that defined categorical exposure, the comparator group comprised participants classified as nonexposed according to the instrument-specific threshold or absence of endorsed trauma domains as reported by the authors. Studies examining CT as a continuous exposure within a single cohort were eligible when they reported association estimates between CT severity scores and metabolic outcomes.

### Data extraction

A structured data extraction table was used to systematically collect and organize data. Three reviewers (NM, IO, GG) independently conducted data extraction on included studies. Information to be extracted was established a priori and included: (1) the first author, year of publication, and study type, (2) title, (3) sample size, (4) outcomes of interest, and (5) findings. All results that examined the association (odd ratios [OR], beta-coefficients, variance, etc.) between childhood trauma and metabolic markers were considered an outcome of interest.

### Quality assessments

Studies were critically appraised by three independent reviewers (VV, AU, AM) using the established tools provided by the National Institutes of Health (NIH), including the Quality Assessment of Controlled Intervention Studies, the Quality Assessment of Observational Cohort and Cross-Sectional Studies, the Quality Assessment of Case–Control Studies, and the Quality Assessment Tool for Before–After (Pre–Post) Studies with No Control Group Studies.^25^^,^^26^ Detailed risk of bias assessment tables are available in Tables S2 and S3 in the Supplementary Material. Quality ratings were not used as exclusion criteria; instead, they were used to contextualize the narrative synthesis, to highlight potential sources of bias when interpreting individual findings, and to inform the strength of inferences across metabolic domains.

## Results

### Search results

Key characteristics of the included studies are summarized in Table 2. Overall, 12 studies were cross-sectional, 2 were case–control, and 2 were prospective cohort studies. Across studies, CT exposure was most commonly assessed using the CTQ/CTQ-SF measures, with some studies examining CT as a categorical exposure (exposed vs. non-exposed) and others modeling CT severity scores continuously. Metabolic outcomes spanned adiposity indices (BMI and waist circumference), lipid fractions and related indices (eg, HDL, LDL, triglycerides, atherogenic index of plasma), glycemic markers (HbA1c/glucose), and inflammatory markers (CRP/hs-CRP).

**Table 2.** Characteristics and Outcomes of the Studies Included in the Systematic Review

Author (year of publication) and study type	Title	Sample size	Outcomes of interest	Findings
Guenzel et al. (2016)^28^ Cross sectional	Adverse events in childhood as a risk factor for elevated BMI among people with schizophrenia and bipolar disorder	299	BMI	Subjects who had experienced physical abuse from a parent had a higher risk for obesity than those who did not (*p* = 0.003, OR = 2.77). Subjects who experienced emotional neglect from their fathers had a higher risk of obesity (*p* < 0.01, OR = 2.59).
Fischer et al. (2021)^37^ Cross sectional	Assessing the links between childhood trauma, C-reactive protein, and response to antidepressant treatment in patients with affective disorders	129	BMI Hs-CRP	Hs-CRP levels in the study population did not differ significantly from the healthy controls (*r* = 0.05, *p* = 0.810). Sexual abuse (β = 0.330, *p* = 0.007) and increased BMI (β = 0.427, *p* = 0.000) showed a significant positive association.
Liboni-Cavicchioli et al. (2018)^27^ Cross sectional	Associations between severity of anxiety and clinical and biological features of major affective disorders	163	HDL	HDL-cholesterol had an inverse relation with all subtypes of childhood trauma (*F* = 12.66, *p* = 0.001)
Godin et al. (2021)^36^ Cross sectional	Childhood maltreatment and metabolic syndrome in bipolar disorders in search of moderators	2,390	Atherogenic index of plasma (AIP) BMI Disposition index (DI) Hs-CRP Total cholesterol	Association between a higher CTQ score, hypertriglyceridemia (*p* = 0.03), and a higher AIP (*p* = 0.05) was identified. The associations between the CTQ total score and other indices related to a higher cardiovascular risk (abdominal obesity, body mass index, abnormal CRP, and DI) were not significant.
Aas et al. (2017)^35^ Cross sectional	Childhood maltreatment severity is associated with elevated C-reactive protein and body mass index in adults with schizophrenia and bipolar diagnoses	483	BMI Glycoprotein 130 Hs-CRP	Patients with childhood maltreatment had increased levels of hs-CRP (*p* < 0.001, Cohen’s *d* = 0.4), lower levels of gp130 (*p* < 0.001, Cohen’s *d* = 0.5), and higher BMI (*p* < 0.001, Cohen’s *d* = 0.5) than the HC group. Up to three types childhood trauma (sexual abuse, physical abuse, and emotional abuse) was associated with elevated BMI (*F* = 8.46, *p* < 0.001, Cohen’s *d* = 0.5) and hs-CRP (*F* = 5.47, *p* = 0.001, Cohen’s *d* = 0.3).
Alciati et al (2011)^34^ Cross sectional	Childhood parental loss and bipolar spectrum in obese bariatric surgery candidates	120	BMI	The subjects who had lost a parent during childhood due to death or separation had significantly higher BMI values than the obese subjects raised in an intact family (*t* = −2.09, df = 117, *p* = 0.03). The obese bipolar II subjects reported having experienced childhood parental loss significantly more frequently (45.3%; 24 patients) than the normal weight bipolar II controls (20%; 11 subjects): odds ratio 3.31; 95% CI: 1.41–7.78
Congio et al. (2022)^32^ Cross sectional	Childhood trauma, interleukin–17, C-reactive protein, metabolism, and psychosocial functioning in bipolar depression	167	BMI Glucose HDL Hs-CRP IL17 IL–1β Leptin Triglycerides Waist circumference	There was a significantly positive correlation between child sexual abuse and BMI, hs-CRP and waist circumference in patients with BD who were currently in a major depressive episode compared to the correlations in the controls and remitted BD groups (*p* < 0.01). There was no significant correlation between childhood trauma and BMI, glucose, HDL, hs-CRP, IL17, IL–1β, leptin, triglycerides, and waist circumference in patients remitted BD groups compared to the correlations in the controls.
Monteleone et al. (2020)^31^ Cross sectional	Clinical and neuroendocrine correlates of childhood maltreatment history in adults with bipolar disorder	106	BMI Cortisol awakening response (CAR)	Childhood trauma patients showed a significant higher body mass index (*p* = 0.01). CAR levels were significantly higher in patients without childhood trauma compared to those with it (*p* = 0.01).
Alciati et al. (2016)^30^ Cross sectional	Psychiatric disorders and childhood parental loss in obesity: Relationship with the mode of weight gain	140	BMI	Subjects with sudden weight gain had a higher prevalence of bipolar II disorder than those with gradual weight gain (χ^2^ = 13.64, *p* < 0.001, OR = 2.77). Childhood parental death was more common in the sudden weight gain group (χ^2^ = 8.076, *p* = 0.004, OR = 5.6), as was parental separation (χ^2^ = 9.906, *p* = 0.002, OR = 2.59). Conversely, pure hypomania was more frequent in the gradual weight gain group (χ^2^ = 13.64, *p* < 0.001, OR = 3.2).
McIntyre et al. (2012)^38^ Cross sectional	The association between childhood adversity and components of metabolic syndrome in adults with mood disorders: Results from the international mood disorders collaborative project	373	BMI BP Fasting glucose HDL Waist circumference	Among subjects with a history of sexual abuse, a significant proportion had a BMI ≥ 30 kg/m^2^, (45.28% vs. 32.88%; *p* = 0.010). Individuals reporting a history of physical abuse, parental loss, or any childhood trauma had higher systolic and diastolic blood pressure (systolic: *p* = 0.040; diastolic: *p* = 0.038), when compared to subjects without a reported history of childhood trauma. Lower glucose levels were identified among subjects with a history of parental loss (*p* = 0.035) and sexual abuse (*p* = 0.038). Lower levels of HDL cholesterol were identified in subjects with a history of parental loss (*p* = 0.024) and any childhood trauma (*p* = 0.021), with a trend among subjects with physical abuse (*p* = 0.060). A trend toward higher waist circumference was identified among subjects with a history of physical abuse (*p* = 0.077).
Alciati et al. (2013)^39^ Cross sectional	The relationship between childhood parental loss and metabolic syndrome in obese subjects	135	BMI	Subjects who had experienced physical abuse from a parent had a higher risk for obesity than those who did not (*p* = 0.003, OR = 2.77). Subjects who experienced emotional neglect from their fathers had a higher risk of obesity (*p* < 0.01, OR = 2.59). Among females with psychiatric disorders, those who had been physically abused by a parent were at a higher risk for obesity (*p* = 0.003, OR = 3.34). Additionally, females who reported emotional neglect from their fathers had a higher risk of obesity (*p* < 0.042, OR = 2.44).
Khosravani et al. (2025)^40^ Cross sectional	Psychobiological mechanisms involved in suicidal risk, suicide attempts, and aggression: A replication and extension in bipolar disorder	353	CRP Total cholesterol Triglycerides LDL HDL TSH FT3 FT4	Compared to individuals without a history of childhood maltreatment, those with such a history presented higher levels of CRP (*F* = 58.21, *p* < 0.001) and lower levels of TC (*F* = 36.62, *p* < 0.001) and HDL-C (*F* = 68.79, *p* < 0.001), with effect sizes ranging from medium (for TC: η HDL-C: η^2^ = 0.10) to large (for CRP: η^2^ = 0.15 and = 0.17). No significant differences were found between the groups with respect to other markers (*p* > 0.05).
Leclerc et al. (2018)^29^ Case–control	The differential association between history of childhood sexual abuse and body mass index in early and late stages of bipolar disorder	152	BMI	The HC group had a mean BMI of 26.88 kg/m^2^, similar to the early-BD subgroup, but significantly different than late-BD (mean 29.93 kg/m^2^). In the same way, the BMI of early-BD significantly differed from the late-BD (*p* = 0.001).
Congio et al. (2022)^41^ Case–control	Cognitive impairment, childhood trauma, sedentary behavior, and elevated C-reactive protein levels in major affective disorders	109	Hs-CPR	BD patients with hs-CRP ≥ 5 mg/L levels presented higher scores on CTQ and reported more emotional abuse, emotional neglect and physical neglect (*p* = 0.01).
Lunding et al. (2021)^33^ Cohort	Childhood trauma and cardiometabolic risk in severe mental disorders: the mediating role of cognitive control	819	BMI Cholesterol total HDL Triglycerides Waist circumference	Experience of three or more subtypes of childhood trauma was positively associated with waist circumference in patients with SMDs (B = 3.145; *t* = 2.459; *p* = 0.014). There were no significant associations between CTQ variables and lipid or adiposity measures (cholesterol total, HDL, BMI and triglycerides) in the total sample.
Galvez-Florez et al. (2025) Cohort	Childhood trauma exposure associated with a more severe course of metabolic disturbances in bipolar disorder: A 36-month longitudinal prospective cohort study	305	BMI HbA1c Hs-CRP HDL LDL Total cholesterol Triglycerides	Childhood trauma was significantly associated with a higher incidence and greater severity of metabolic disturbances over time, with large effect sizes. HbA1C: LS mean difference 1.11; *p* < 0.001; Cohen’s *d* = 1.11 BMI: LS mean difference 4.46; *p* < 0.001; Cohen’s *d* = 1.86 Total cholesterol: LS mean difference 37.67; *p* < 0.001; Cohen’s *d* = 3.17 HDL: LS mean difference − 5.63; *p* < 0.001; Cohen’s *d* = 1.21 LDL: LS mean difference 30.18; *p* < 0.001; Cohen’s *d* = 2.51 Triglycerides: LS mean difference 33.72; *p* < 0.001; Cohen’s *d* = 0.97 Hs-CRP: LS mean difference 2.68; *p* < 0.001; Cohen’s *d* = 4.03

The initial literature search generated 425 studies. After removing 148 duplicates, 277 studies underwent title and abstract screening, of which 32 were eligible for full-text screening, as outlined on the PRISMA flowchart (Figure 1). Most of the previous studies were discarded as they did not meet the inclusion criteria. Finally, from full-text screening, 16 eligible publications were included in the systematic review, the majority of which were excluded due to full text not available (*n* = 1), nonprimary research (*n* = 8), an ineligible outcome (*n* = 4), and an ineligible population (*n* = 2).

#### Quality assessment

The methodological quality of the included studies was evaluated using the NIH Quality Assessment Tools for observational and case–control designs (Tables S2 and S3 in the Supplementary Material). Among the 16 studies included, most of cross-sectional and cohort studies were rated as having fair methodological quality (68.75%), while only 31.25% met criteria for a good quality rating. Common limitations included lack of adjustment for key confounders (eg, psychotropic medication use, illness duration), incomplete reporting of follow-up rates, and limited use of standardized trauma assessments across studies. For case–control studies, only one study ^27^ received a good quality rating, while the remainder were classified as fair due to concerns such as unclear selection procedures, insufficient control for covariates, or incomplete blinding of outcome assessment. Despite these methodological constraints, the overall quality was sufficient to support the synthesis of evidence across multiple metabolic domains. However, the variability in study quality underscores the need for future investigations to adopt more robust designs, standardized exposure and outcome assessments, and comprehensive reporting in accordance with contemporary epidemiological standards.

#### Qualitative findings

##### Childhood trauma, BMI, and BD

BMI was assessed in 13 (81.3%) of the 16 included studies. Overall, 11 (84.6%) studies reported a statistically significant positive association between CT exposure and higher BMI or obesity risk, whereas 2 (15.4%) studies reported no significant association. Cross-sectional investigations provided most of the evidence: among the 10 cross-sectional studies assessing BMI, 9 reported significantly higher BMI or increased odds of obesity in participants with histories of childhood abuse/neglect, whereas one study reported null findings. In a case–control study, Leclerc et al. reported higher BMI among individuals with BD and childhood sexual abuse, particularly in later stages of illness. Findings from prospective cohorts were mixed. Galvez-Florez et al. observed a larger BMI increase over 36 months in CT-exposed BD participants (mean difference ≈ +4.5 BMI units; *p* < 0.001; Cohen’s *d* = 1.86), whereas Lunding et al. did not detect significant associations between CT exposure and BMI over follow-up. Collectively, these findings support a consistent association between CT exposure and higher BMI in BD, while highlighting the need for additional prospective studies.

##### Childhood trauma, lipid profile, and BD

Across the studies included, seven investigations assessed lipid profiles and triglyceride levels in relation to CT exposure among individuals with BD. Among these, five studies (71.4%) reported significant or positive associations between CT and adverse lipid parameters, including elevated triglyceride levels, increased atherogenic index of plasma, and reduced HDL cholesterol concentrations. In contrast, two (28.6%) studies did not identify statistically significant relationships between CT exposure and lipid markers. Specifically, Godin et al. observed associations between higher CTQ total scores and hypertriglyceridemia, as well as elevated AIP, whereas McIntyre et al. reported lower HDL cholesterol levels among participants with a history of parental loss and physical abuse. Khosravani et al. found that individuals with CT exposure had lower total cholesterol and HDL and these lipids abnormalities were associated in the participants with suicide attempts. Conversely, Congio et al. found no significant correlations between CT exposure and lipid markers in remitted BD participants. Nevertheless, associations were reported during depressive episodes for waist circumference and BMI. Among the two cohort studies, Galvez-Florez et al. demonstrated that CT exposure was associated with significantly higher total cholesterol, LDL cholesterol, and triglyceride levels over a 36-month follow-up, with large effect sizes, highlighting the cumulative cardiometabolic burden associated with CT exposure in BD.

##### Childhood trauma, glycosylated hemoglobin, and BD

Only one study assessed glycosylated hemoglobin (HbA1c) in relation to CT exposure among individuals with BD. In the 36-month prospective cohort study by Galvez-Florez et al., CT exposure was associated with higher HbA1c over follow-up, with large effect sizes. Evidence on other glycemic markers was limited and mixed; for example, Congio et al. reported no significant association between CT exposure and fasting glucose in remitted BD participants, though associations with anthropometric indices were observed during depressive episodes. Overall, the available data suggest a potential association between CT exposure and glycemic dysregulation in BD, but additional longitudinal studies are needed.

##### Childhood trauma, C-reactive protein, and BD

Seven studies evaluated the association between CT exposure and circulating levels of C-reactive protein (CRP/hs-CRP) in individuals with BD. Overall, five studies reported statistically significant associations between CT exposure (or specific CT subtypes) and higher CRP/hs-CRP, whereas two studies reported null findings in certain clinical states. Aas et al. reported elevated hs-CRP among individuals with childhood maltreatment. Galvez-Florez et al. observed markedly higher hs-CRP over 36 months among CT-exposed participants, with very large effect sizes. Fischer et al. reported a positive association between childhood sexual abuse and hs-CRP, and Congio et al. found higher CTQ scores among BD participants with hs-CRP ≥5 mg/L, with stronger associations for emotional abuse/neglect domains. Khosravani et al. reported higher CRP among individuals with childhood maltreatment. Conversely, some studies did not identify significant differences in CRP/hs-CRP between CT-exposed and nonexposed groups during euthymic or remitted states. Taken together, the evidence supports an association between CT exposure and elevated inflammatory burden in BD, particularly during symptomatic phases.

## Discussion

This is the first systematic review that provides a comprehensive synthesis of the association between CT exposure and metabolic disturbances in the course of bipolar illness. Our findings suggest that CT exposure is consistently associated with metabolic adversity. We have documented altered metabolic disturbances such as elevated body mass index (BMI), positive inflammatory markers such as high-sensitivity C-reactive protein (hs-CRP), and dysregulated lipid profiles. In other words, our results highlight the clinical relevance of CT exposure as a course modifier for both psychiatric and physical health outcomes in BD.

BMI was the most frequently assessed metabolic marker, evaluated in 13 of the 16 included studies. Most studies reported an association between CT exposure and higher BMI or greater obesity risk. Evidence was strongest in cross-sectional designs, where the majority of studies assessing BMI reported higher adiposity among CT-exposed participants. Cohort findings were more heterogeneous: Galvez-Florez et al. observed a substantial BMI increase over 36 months in CT-exposed BD participants (Cohen’s *d* = 1.86), whereas Lunding et al.^33^ did not detect a significant association, underscoring the need for additional prospective research using standardized trauma assessments.

Regarding lipid metabolism, two third of the studies evaluating triglycerides and cholesterol profiles reported significant associations between CT exposure and adverse lipid parameters, including increased triglyceride levels, elevated atherogenic index of plasma, and decreased HDL cholesterol. These findings are consistent with previous literature that linked early life stress with disruption of lipid homeostasis that may produce chronic inflammatory activation and dysregulated autonomic nervous system responses.^42^^–^^46^ In that sense, Galvez-Florez et al. reported large effect sizes for LDL, total cholesterol, and triglycerides over time, suggesting that early adversity such as CT exposure may contribute to persistent dyslipidemia in BD.

Inflammation emerged as a robust correlate of CT exposure in BD participants, with four of five studies reporting elevated hs-CRP levels among trauma-exposed individuals. These results align with previous meta-analyses showing a dose–response relationship between CT exposure severity and proinflammatory cytokines.^47^ In fact, the association between CT exposure and hs-CRP was particularly pronounced during acute affective episodes, suggesting that inflammation may be state dependent and interact with mood-related physiological processes.^48^^–^^51^ Furthermore, findings from longitudinal studies such as Galvez-Florez et al. strongly support the temporal stability of inflammatory dysregulation, which may serve as a potential biomarker of illness severity and treatment response.

Evidence regarding glycemic markers was limited. Only one longitudinal study reported higher HbA1c levels in BD participants with CT exposure, and data on glucose metabolism remain scarce. While these findings are consistent with hypotheses linking early adversity to insulin resistance via sustained activation of stress–response systems, additional longitudinal studies are required to clarify temporality and confounding in BD populations.

Taken together, the evidence suggests that CT exposure is associated with a metabolic phenotype in BD characterized by increased adiposity, systemic inflammation, and lipid abnormalities. These alterations may be clinically relevant because they could co-occur with greater psychiatric symptom burden, elevated cardiovascular risk, and poorer treatment outcomes; however, the predominance of observational designs limits causal inference. Accordingly, these findings support trauma-informed metabolic screening in BD and motivate prospective studies designed to disentangle temporality and confounding.

Future research should aim to address current limitations by incorporating longitudinal designs, harmonized CT measurements (eg, CTQ-SF), and multimodal biomarker panels, including cytokine profiling, neuroimaging, and genomic data. Additionally, studies investigating potential moderating variables such as age of trauma exposure, trauma subtypes, sex differences, and psychotropic medication use will be essential to disentangle the complex biopsychosocial interactions underlying these associations.

This systematic review brings into consideration important clinical and translational implications for BD intervention. It emphasizes the need to incorporate a trauma-informed framework into the assessment and treatment of BD. This means being able to perform CT screening using standardized psychometric instruments such as the CTQ. On the other hand, metabolic parameters should also be assessed from the beginning of therapeutic intervention and throughout the course of illness. This is true regardless of whether BD participants are prescribed or not with atypical antipsychotics or other medications that might be associated with metabolic disturbances, such as mirtazapine, amitriptyline, or pregabalin. This novel approach will take into clinical consideration both psychological burden and elevated cardiometabolic risk. We argue that consistent associations between CT exposure and altered metabolic markers such as BMI, dyslipidemia, and systemic inflammation suggest that a subgroup of trauma-exposed participants with BD may benefit from integrated interventions that address both mental and physical health domains right from the beginning of treatment. In other words, behavioral and pharmacological interventions targeting metabolic dysregulation, including anti-inflammatory agents, lifestyle modification programs, and structured weight management may offer synergistic benefits when applied early in trauma-exposed BD participants. In this sense, early prevention and public health strategies focused on reducing childhood adversity to mitigate long-term psychiatric and metabolic consequences is warranted.

Additionally, the exclusive focus on BD in this review is supported by emerging evidence that individuals with BD may present a distinct pattern of metabolic and inflammatory dysregulation, with a particular gap in the context of CT exposure. Recent findings suggest that incorporating metabolic biomarkers into diagnostic models enhances the ability to differentiate BD from major depressive disorder (MDD), especially in individuals presenting with depressive episodes or ambiguous symptoms.^52^ This added diagnostic value is especially pronounced when psychiatric symptom data are incomplete or unavailable, highlighting the potential utility of biomarker profiles in clinical decision making. Furthermore, several biomarkers reviewed such as hs-CRP, glucose, and lipid fractions have demonstrated stronger correlations with manic symptom dimensions, suggesting they may reflect state-dependent or trait-level physiological alterations in BD.^53^ While direct comparisons across diagnostic categories remain limited,^54^ these preliminary findings support the biological plausibility of BD-specific vulnerability to trauma-related metabolic alterations. Nonetheless, we recognize this as a critical knowledge gap and a priority for future comparative research to delineate disorder-specific versus transdiagnostic effects.

Strengths of this systematic review include a rigorous methodology adhering to PRISMA 2020 guidelines, a comprehensive multidatabase search strategy, and clearly defined inclusion criteria focusing specifically on BD populations. Furthermore, our work integrates both cross-sectional and longitudinal data, allowing for a more nuanced understanding of temporal associations. The inclusion of multiple metabolic domains, ranging from BMI to lipid profiles, HbA1c, and inflammatory markers also strengthens the ecological validity of the findings, capturing the multifaceted impact of CT exposure on systemic physiology.

Several limitations must be acknowledged. First, the heterogeneity of included studies in terms of design, trauma assessment methods in the assessment of childhood trauma across studies. While some investigations utilized validated and widely recognized instruments such as the Childhood Trauma Questionnaire Short-Form (CTQ-SF), others relied on nonstandardized self-report measures, clinical interviews, or retrospective chart reviews. These differences not only vary in terms of content coverage, ranging from emotional, physical, and sexual abuse to neglect and household dysfunction, but also in the depth, scoring systems, and thresholds used to define trauma exposure or severity. This variability complicates direct comparisons between studies and introduces potential classification bias, as the same individual might be identified as trauma exposed in one study and nonexposed in another depending on the instrument used. Furthermore, the retrospective nature of trauma assessment may be subject to recall bias, particularly in populations with mood instability or cognitive impairment. Collectively, this heterogeneity undermines the consistency and reliability of trauma classification and represents a critical challenge in quantitative synthesis of the literature on childhood trauma and metabolic biomarkers in bipolar disorder. This issue limited the possibility of conducting a meta-analysis and may reduce the comparability of findings. Second, most of included studies were cross-sectional, precluding causal inference and limiting insight into temporal dynamics between CT exposure and metabolic dysregulation. Although two prospective cohort studies were identified, findings were inconsistent and warrant replication. Third, residual confounding due to unmeasured variables such as psychotropic medication effects, diet, sleep quality, and socioeconomic status could not be ruled out in most studies. Ultimately, publication bias remains a concern, as studies reporting null associations may be underrepresented in the published literature.

## Conclusion

This systematic review underscores a consistent and clinically meaningful association between CT exposure and adverse metabolic outcomes in individuals with BD. Across diverse study designs, CT exposure was linked to increased BMI, dyslipidemia, glycemic dysregulation, systemic inflammation, and metabolic alterations that may exacerbate psychiatric morbidity and elevate cardiovascular risk. These findings highlight the need for trauma-informed approaches in the clinical management of BD, while integrating psychiatric and metabolic care.

In clinical practice, trauma-informed metabolic screening could involve the routine use of validated childhood trauma assessments such as the Childhood Trauma Questionnaire combined with standard metabolic monitoring, including measurements of BMI, fasting glucose, lipid profiles, and inflammatory markers. This integrated approach is in line with recommendations from the Task Force for the management of patients with mood disorders and comorbid metabolic disorders proposed by The Canadian Network for Mood and Anxiety Treatments (CANMAT),^21^ and the American Heart Association for managing cardiometabolic risk in patients with mood disorders.^3^ Incorporating trauma history into metabolic risk evaluations may enhance early identification of high-risk individuals and guide personalized interventions in bipolar disorder care.

Furthermore, the biological imprint of early adversity observed in these studies suggests that CT exposure contributes to a distinct cardiometabolic phenotype within the bipolar spectrum. Addressing this comorbidity early through routine trauma screening, metabolic monitoring, and multidisciplinary interventions may reduce long-term disability and improve functional outcomes. Future research should prioritize longitudinal, multimodal investigations to delineate causal pathways and identify modifiable targets for early intervention. Multimodal efforts are critical to advance into personalized medicine in psychiatry and thus mitigating the compounding burden of mental and physical illness in BD.

## Supporting information

10.1017/S1092852926100881.sm001 Guillen-Burgos et al. supplementary material Guillen-Burgos et al. supplementary material

## Data Availability

The data that support the findings of this study are available from the corresponding author, upon reasonable request.

## Supplementary material

The supplementary material for this article can be found at http://doi.org/10.1017/S1092852926100881.
