# Supplementary material for: Metabolic markers in bipolar disorder with childhood trauma exposure: a systematic review
Source: CNS Spectr. 2026 Mar 24;31(1):e8. doi: 10.1017/S1092852926100881 (PMC13076081; doi:10.1017/S1092852926100881)
Supplement: Guillen-Burgos et al. supplementary material [file S1092852926100881sup001.docx]

**Supplementary material**

**Table S1. Search string implemented for each database.**

| **Concept** | **Search String*** |
| --- | --- |
| Bipolar disorder | "bipolar disorder" OR "bipolar and related disorders" OR "mood disorder bipolar" OR "affective psychosis bipolar" OR "bipolar affective psychosis" OR "psychoses bipolar affective" OR "psychoses manic depressive" OR "manic depressive psychosis" OR "depression bipolar" OR "bipolar depression" OR "manic disorder" OR "bipolar disorder type 1" OR "type 1 bipolar disorder" OR "bipolar disorder type 2" OR "type 2 bipolar disorder" OR "bipolar affective disorder" OR "bipolar and related disorders" OR "bipolar illness" OR "bipolar psychosis" OR "depression, manic" OR "manic depression" OR "manic depression psychosis" OR "manic depressive" OR "manic depressive disease" OR "manic depressive disorder" OR "manic depressive illness" OR "manic depressive reaction" OR "manic depressive syndrome" OR "manic depressive psychosis" OR "manic depressive syndrome" OR "psychosis, manic depressive" |
| Childhood trauma | "childhood trauma" OR "adverse childhood experiences" OR "child abuse" OR "child abuse, sexual" OR "childhood experience adverse" OR "early life stress" OR "life stress early" OR "stress early life" OR "childhood trauma questionnaire" OR "childhood trauma exposure" OR "trauma childhood" OR "traumatic childhood experiences" OR "childhood experiences traumatic" OR "experiences traumatic childhood" OR "adolescent trauma" OR "abuse child" OR "childhood maltreatment" OR "maltreatment child" OR "child mistreatment" OR "child neglect" OR "neglect child" OR "child neglect experiences" OR "experience childhood neglect" OR "childhood abuse experience" OR "experience childhood abuse" OR "physical neglect childhood" OR "childhood physical neglect" OR "child neglect physical" OR "neglect physical child" OR "physical child neglect" OR "child emotional abuse" OR "emotional abuse" OR "sexual abuse" OR "child sexual abuse" OR "child trauma" OR "childhood traumatic experience" OR "childhood traumatic experiences" OR "childhood traumatic stress" OR "paediatric trauma" OR "pediatric trauma" OR "pediatric traumatic stress" |
| Metabolic biomarkers | "body mass index" OR "bmi" OR "glucose" OR "glycosylated hemoglobin" OR "hb1ac" OR "glycated hemoglobin" OR "cholesterol" OR "total cholesterol" OR "high density lipoprotein cholesterol" OR "hdl" OR "low density lipoprotein cholesterol" OR "ldl" OR "triglyceride" OR "triglycerides" OR "c-reactive protein" OR "crp" OR "hs-crp" |

*The search string for each concept was combined with the boolean operator “AND”.

**Table S2. Risk of bias/quality assessment of the included studies using the NIH Quality Assessment of Observational Cohort and Cross-Sectional Studies**

| **Study** | **Item** | | | | | | | | | | | | | | **Quality Rating** |
| --- | --- | --- | --- | --- | --- | --- | --- | --- | --- | --- | --- | --- | --- | --- | --- |
|  | **1** | **2** | **3** | **4** | **5** | **6** | **7** | **8** | **9** | **10** | **11** | **12** | **13** | **14** |  |
| Alciati et al. (2011b) | ✓ | X | ✓ | ✓ | X | X | X | ✓ | ✓ | NA | ✓ | NA | NA | ✓ | Fair |
| Alciati et al. (2015) | ✓ | ✓ | NR | ✓ | X | X | X | ✓ | ✓ | NA | ✓ | NA | NA | X | Fair |
| Galvez et al. (2025). | ✓ | ✓ | CD | ✓ | ✓ | ✓ | ✓ | ✓ | ✓ | X | ✓ | NA | NA | ✓ | Good |
| Godin et al. (2021) | ✓ | ✓ | NR | ✓ | ✓ | X | X | ✓ | ✓ | NA | ✓ | NA | NA | ✓ | Good |
| Khosravani et al. (2025) | ✓ | ✓ | ✓ | ✓ | X | X | X | ✓ | ✓ | X | ✓ | X | ✓ | ✓ | Fair |
| Leclerc et al. (2017) | ✓ | X | CD | CD | ✓ | X | X | ✓ | ✓ | NA | ✓ | NA | NA | ✓ | Fair |
| McIntyre et al. (2012) | ✓ | ✓ | ✓ | ✓ | X | X | X | ✓ | ✓ | NA | ✓ | NA | NA | ✓ | Good |
| Monteleone et al. (2020) | ✓ | X | ✓ | ✓ | ✓ | X | X | ✓ | ✓ | NA | ✓ | NA | NA | ✓ | Good |

Symbols: ✓- yes; X - no

Abbreviations: NR = not reported; NA = not applicable; CD = cannot determine

**Table S3. Risk of bias/quality assessment of the included studies using the NIH Quality Assessment of Case-Control Studies**

| **Study** | **Item** | | | | | | | | | | | | **Quality Rating** |
| --- | --- | --- | --- | --- | --- | --- | --- | --- | --- | --- | --- | --- | --- |
|  | **1** | **2** | **3** | **4** | **5** | **6** | **7** | **8** | **9** | **10** | **11** | **12** |  |
| Aas et al. (2017) | ✓ | ✓ | X | ✓ | ✓ | ✓ | NA | ✓ | NA | X | NA | X | Fair |
| Alciati et al. (2011a) | ✓ | X | X | ✓ | X | ✓ | NA | ✓ | NA | ✓ | NA | ✓ | Fair |
| Cavicchioli et al. (2017) | ✓ | ✓ | X | ✓ | ✓ | ✓ | NA | ✓ | NA | ✓ | NA | ✓ | Good |
| Congio et al. (2022a) | ✓ | X | X | ✓ | ✓ | ✓ | NA | ✓ | NA | ✓ | NA | ✓ | Fair |
| Congio et al. (2022b) | ✓ | ✓ | X | CD | ✓ | ✓ | NA | ✓ | NA | ✓ | NA | ✓ | Fair |
| Fischer et al. (2021) | ✓ | X | X | CD | ✓ | ✓ | NA | ✓ | NA | ✓ | NA | ✓ | Fair |
| Guenzel et al. (2016) | ✓ | ✓ | X | ✓ | ✓ | ✓ | NA | ✓ | NA | ✓ | NA | X | Fair |
| Maes et al. (2018) | ✓ | X | X | ✓ | ✓ | ✓ | NA | ✓ | NA | ✓ | NA | ✓ | Fair |

Symbols: ✓ - yes; X - no

Abbreviations: NR = not reported; NA = not applicable; CD = cannot determine
